# Progress in GABAA receptor agonists for insomnia disorder

**DOI:** 10.3389/fphar.2024.1432726

**Published:** 2024-11-05

**Authors:** Weiyi Wang, Wanting Fu, Hongyan Zhu, Jing Ma, Jian Zhang, Jia Qi

**Affiliations:** Department of Clinical Pharmacy, Xinhua Hospital Affiliated to Shanghai Jiaotong University Affiliated to School of Medicine, Shanghai, China

**Keywords:** insomnia, GABAA receptor, benzodiazepines binding site, dimdazenil, partial positive allosteric modulator

## Abstract

Insomnia is the most common sleep disorder in which an individual has trouble falling or staying asleep. Chronic sleep loss interferes with daily functioning and adversely affects health. The main clinical drugs for insomnia are the positive allosteric modulator of the GABA (gamma-aminobutyric acid) A receptors (GABAARs) at the benzodiazepine site with selectivity of the GABA-α1 receptor. They are divided into benzodiazepine drugs and non-benzodiazepine drugs. Most recently, the first partial positive allosteric modulator of GABAAR Dimdazenil was approved by National Medical Products Administration (NMPA) and launched in China. This review summarized the mechanism of actions of current clinical drugs for insomnia, and the clinical applications of these drugs, which may help to understand their involvement in insomnia, and to search for more selective and potent ligands to be used in the treatment of insomnia.

## 1 Introduction

Insomnia is a symptom characterized by difficulty with sleep onset and/or sleep maintenance, which is defined by key criteria including insomnia symptoms along with daytime symptoms occurring at least three times per week for at least 3 months (Riemann et al., 2020). According to this definition, as long as daytime function is impaired, every sleep complaint (whether related to sleep quality or sleep quantity) is sufficient to diagnose insomnia (Morin et al., 2015; St Louis and Boeve, 2017). There is an increasing number of people suffering from insomnia problems such as difficulty falling asleep, early awakening, and short sleep duration currently due to the social and economic pressures of modern societies which COVID-19 intensified. Insomnia is associated with impaired daytime functioning, cognitive function, and occupational performance, resulting in irritability, anxiety, fatigue, inattention, memory impairment, and increased reaction time (Luzzi et al., 2022; Kantrowitz et al., 2009). Elder people are particularly vulnerable to insomnia, with nearly half of people over 65 estimated to suffer from insomnia (Proserpio et al., 2022; Lou and Oks, 2021). These symptoms are especially concerning in older adults, who may have already experienced cognitive decline from aging or other conditions (Saletu et al., 2005). Insomnia is linked to the development of Alzheimer’s disease (AD), with aging being the biggest risk factor for late-onset AD (Wu et al., 2010). Chronic insomnia may seriously affect our work and life, and increase the risk of physical and mental diseases, resulting in various economic and social burden. With the increasing incidence of insomnia and the growing self-awareness of patients, the demand for hypnotics increased gradually. Presently, the main clinical drugs for insomnia are the positive allosteric modulator of the GABA (gamma-aminobutyric acid) A receptors (GABAARs) at the benzodiazepine site with selectivity of the GABA-α1 receptor, most of which have certain adverse reactions. Recently, Dimdazenil, the first partial positive allosteric modulator of GABAAR, received approval from the National Medical Products Administration (NMPA) and was launched in China. This review outlines the mechanisms of action and clinical applications of current insomnia medications. Understanding these aspects may provide insights into their role in treating insomnia and guide the search for more selective and effective ligands for future therapies.

## 2 Underlying mechanisms of GABAA receptor agonists in insomnia

The regulation of the sleep-wake cycle is complex involving multiple brain circuits and signal pathways. On one hand, interactions between a number of neuroanatomical and neurochemical systems, including acetylcholine, norepinephrine, dopamine, serotonin, histamine, and hypocretin (orexin), have been shown to control waking state (Brown et al., 2012; Schwartz, 2011). On the other hand, sleep onset is controlled by the activity of sleep-promoting neurons located in the anterior part of the hypothalamus, which utilizes GABA to inhibit areas that promote wakefulness (Murillo-Rodriguez et al., 2012).

**FIGURE 1:**
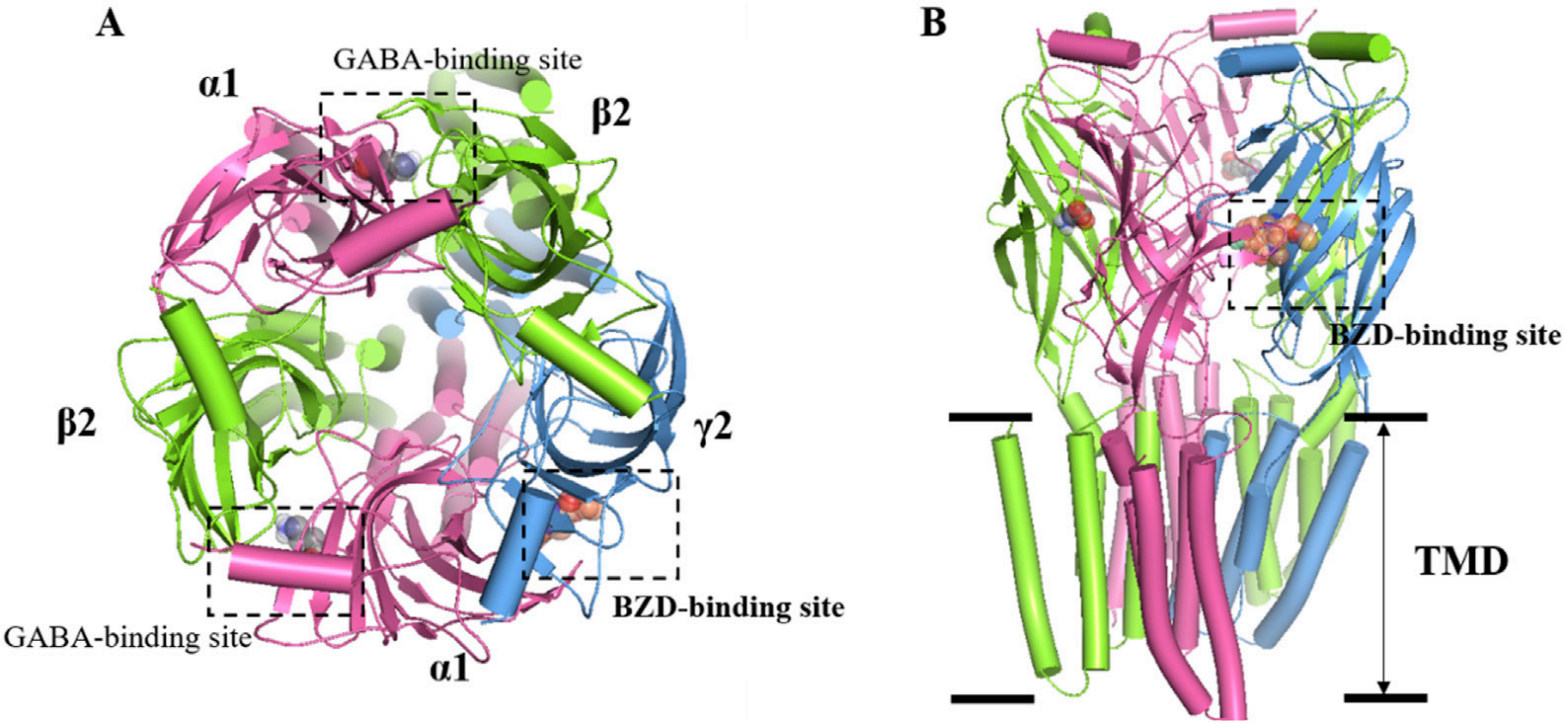
Overall structure of the GABAA receptor. **(A)** Top view: two GABAs binding sites are found at the junctions between α1 and β2 subunits, while the BZDs is located at the interface of α1 and γ2. α1 subunit is colored in pink, while γ2 is blue and β2 is green. **(B)** Side view: the heteropentameric arrangement of subunits in the 2:2:1 stoichiometry of α, β, and γ subunits that comprise the most abundant form of GABAAR, arranged around the central chloride-permeable channel pore.

GABA is the main inhibitory neurotransmitter in the brain. It plays a crucial inhibitory role in the central nervous system, and regulates neural activity and emotions. GABA promotes relaxation and sleep by inhibiting the excitability of neurons and reducing neurotransmission and activity. In the brain, GABA exerts its effects primarily through two types of receptors: GABA-A receptors (GABAARs) and GABA-B receptors (GABABRs). GABAARs are a family of ligand-gated chloride anion channels widely expressed in the central nervous system (CNS) and consist of five subunits, each with several isoforms, composed of 19 related isoforms (α1−6, β1−3, γ1−3, δ, ε, θ, π, ρ1−3). The most abundant GABAAR forms consist of α, β, and γ subunits in a 2:2:1 stoichiometry. The binding site for the orthosteric ligand is located at the interface between α and β subunits, resulting in two GABA binding sites per heteropentamer (Figure 1). Studies have shown that the functions of α subunits of GABAA receptors can be generalized as follows: α1 is closely related to sedative function without anti-anxiety effect, but benzodiazepine addiction; α2 and α3 mainly mediate anti-anxiety and muscle relaxation effect without sedative effect; α5 is closely associated with learning and memory processes (Rudolph and Möhler, 2006). In the mammalian brain, there are approximately 25 different subtypes of GABAARs that occur in different subcellular locations. The neurotransmitter GABA is released into a synaptic cleft from the presynaptic nerve terminus when a GABAergic (GABA-releasing) neuron fires. GABA binds to GABAARs in postsynaptic nerve terminus, and changes their conformation state. GABAARs open the pore to allow chloride anion to move through the channels, and lower their electrochemical gradient. When GABA binds to it, GABAAR will increase the opening of chloride anion channels, resulting in increased negative potential in cells. This increase in negative potential inhibits neuronal excitability, producing a calming and anti-anxiety effect. In general, GABA regulates sleep by inhibiting neuronal excitability and reducing neurotransmission and activity, resulting in sedation, relaxation, and anti-anxiety effects (Gottesmann, 2002).

## 3 Current pharmacological therapies and their side effects

### 3.1 Main therapeutic drugs and their mechanisms of action

At present, the marketed medications for insomnia treatment mainly include GABAA receptor positive allosteric modulator, melatonin receptor agonists, antidepressants with hypnotic effects, daridorexant, and other classes (De Crescenzo et al., 2022). At present, the drugs used to treat insomnia in China are still dominated by GABAA receptor positive allosteric modulator at the benzodiazepine site located at the interface of α1 and γ2, also called benzodiazepine receptor agonists (BzRAs) (Figure 1) (Ebert et al., 2006). Both BzRAs with and without the chemical structure of benzodiazepine compounds, which are classed with benzodiazepines (BZDs) and non-benzodiazepines (nBZDs), work by binding at the benzodiazepine site (Berman et al., 2017; Möhler et al., 2002).

BzRAs enhance the effect by binding to the subunits of GABAAR, thereby increasing the opening frequency of the chloride ion channel (Roehrs and Roth, 2012). The occupation of receptors leads to the opening of chloride ion channels, promoting the inhibitory effect of GABA. BzRAs bind to the BZD recognition site of the GABAA receptor as a positive allosteric modulator of the inhibitory neurotransmitter GABA, which means that GABA must also exist on the receptor complex for BzRAs to exert their inhibitory effect. BZDs, such as Diazepam, Lorazepam, and Alprazolam, bind to the α1, α2, α3, and α5 subunits of the GABAAR. BZDs mainly act on the α1 subunits binding site of GABAAR complex for sedation and sleep, and act on the α2 subunits of GABAAR complex for anti-anxiety (Nørgaard et al., 2021; Maramai et al., 2020; Sharkey and Czajkowski, 2008).

### 3.2 Types and side effects of benzodiazepines

BZDs act as widely used sleeping pills in clinical practice. The mechanism of action is to block the impulse conduction from the limbic system to the brain stem reticular structure, reduce the excitatory impulse transmitted from the thalamus to the cerebral cortex, and improve sleep, but do not increase the deep sleep period (Zezula et al., 1988; Nguyen et al., 2013). Due to the influence of the limbic system, BZDs have multiple therapeutic effects such as sedation, hypnotic, antianxiety, anticonvulsant, and muscle relaxation, but cause adverse reactions such as amnesia and addiction.

Noticeably, BZDs do not have precise receptor selectivity, the anti-anxiety, sedation, and hypnotic effects occur at the same time, but also bring muscle relaxation, and affect psychomotor and cognitive function (Da Settimo et al., 2007). When BZDs are used continuously, GABAAR adapts to the sensitivity of benzodiazepine, requiring increased drug doses to achieve the same efficacy. Recently, quite a number of studies have reported that BZDs are addictive and dependent, and long-term usage may cause side effects including excessive sedation, cognitive impairment, consciousness disturbance, withdrawal symptoms, easy falls, fractures, cardiovascular abnormalities, respiratory depression and so on (Crowe and Stranks, 2018; Markota et al., 2016; Puustinen, 2018; Skinner et al., 2017). This makes most guidelines and expert consensus recommend that BZDs should not be used for more than 2–4 weeks (Davies et al., 2017). However, in clinical practice, the duration of use of these drugs is not strictly controlled where BZDs are often used for a long time in the treatment of most anxiety and sleep disorders, and longer when used to improve residual symptoms after first-line treatment (Jørgensen and Osler, 2018; Marsden et al., 2019). Due to the addictive and tolerability issues of the drugs, but given their efficacy many patients are forced to rely on drugs for a long time to maintain sleep. Currently used BZDs also suffer from other limitations, including tolerance, withdrawal symptoms, and ethanol interaction.

There are several types of BZDs including short-acting BZDs such as Midazolam, Triazolam, and Nordazepam with a half-life of about 2–10 h; medium effective varieties such as Lorazepam, Alprazolam, Estazolam, Chlorazepine with a half-life of about 10–24 h; and long-acting varieties such as Diazepam, Nitrazepam, Clonazepam, Flunitrazepam, Flurazepam with a half-life of more than 30 h (Kienitz et al., 2022). Fluazepam, Temazepam, Triazolam, and Midazolam are used in patients who have difficulty falling asleep and who wake up too much or too early at night. Lorazepam is suitable for insomnia in a state of anxiety or temporary, environmental stress.

Both short-term and long-term use of sedative-hypnotics have certain adverse reactions. The rebound and withdrawal symptoms of short-acting drugs are more severe when the drug is stopped. The adverse effects of short-term use of sedative-hypnotic drugs include sedation, vertigo, fatigue, and memory impairment (Gravielle, 2016). Among short-term BZDs users, 15%–44% of patients experienced moderate to severe prolonged withdrawal symptoms, including sudden anxiety and depression (Ashton, 1991). Studies have found that benzodiazepines may affect patients’ cognitive function including memory, attention, perception, and thinking (Kripke, 2016). Case-control studies by French and Canadian researchers have found that long-term use of BZDs may increase the risk of Alzheimer’s disease (Billioti de Gage et al., 2014). Long-term use of sedative-hypnotics will cause psychomotor impairment and memory impairment, and may lead to increased risk of falls (Hajak et al., 2003; Schifano et al., 2019). Patients with long-term use (>6 months) of BZDs will lead to withdrawal symptoms, with 40% exhibiting moderate to severe withdrawal symptoms (Rickels et al., 1990). For patients diagnosed with insomnia for the first time, it is not recommended to choose this type of medication as the first choice. Short-term insomnia should be treated with short-acting varieties, and the duration of medication should not exceed 12 weeks. After discontinuing medication, the dosage should be reduced gradually. For stubborn insomnia, long-acting preparations can be chosen, and various symptoms that the patient may experience during the medication process can be strictly observed, and the dosage can be adjusted in a timely manner. Traditional BZDs, such as Diazepam or Lorazepam, act as full agonists at the BZD recognition site, so one strategy to address the shortcomings of these compounds is to develop partial agonists with lower intrinsic efficacy at the GABAA receptor BZD site (Rundfeldt and Löscher, 2014).

### 3.3 Properties of non-benzodiazepines

For patients with poor therapeutic effect of BZDs or with higher potential risk, clinicians often use nBZDs, such as Zopiclone, Zaleplon, Zolpidem, and Eszopiclone to improve the night sleep in sleep disorders. These so-called “Z-drugs” sedative-hypnotics are often advocated as safer alternatives to BZDs for sleep disturbances as a result of short half-life and preservation of healthy sleep architecture. nBZDs have different binding affinities to different subunits of GABAAR. These differences include that Zolpidem has relatively high affinity for α1 compared with α3 subunit containing GABAA receptors. In addition, nBZDs have strong binding affinity on the α1 subunit of GABAAR, however, weak binding affinity on α2, α3, or α5 subunits, resulting in strong sedative and hypnotic effects but lacking obvious anti-anxiety, anti-epileptic, or muscle relaxation effects (Krystal, 2010). nBZDs affect the same receptor as BZDs, suggesting that their risks may be similar. nBZDs in the treatment of insomnia, compared with traditional BZDs, have the advantages of lower dependence and tolerance, less occurrence of rebound insomnia after withdrawal, and relatively small respiratory depression and muscle relaxation effects (Roth et al., 2005; Hajak et al., 2002; Israel and Kramer, 2002; Zhang et al., 2014). However, there are still side effects, long-term use risks, and withdrawal reactions. Clinical trials of Zolpidem in healthy young people have shown central nervous system side effects, including impaired cognitive and motor function, particularly in the first few hours of use (Troy et al., 2000; Swainston Harrison and Keating, 2005; Frey et al., 2011). Compared to Zaleplon, Zopiclone exhibits higher dependence and overdose-related issues, but slightly lower abuse and withdrawal reactions. Zolpidem, as the most commonly used medication in the Z-drugs, is associated with intravenous injection, high-dose use, and concomitant use of recreational drugs. Compared to Zopiclone and Zaleplon, Zolpidem is more prone to non-medical use, abuse, and discontinuation (Krystal, 2010). The abuse of zolpidem is also associated with an increased risk of delusions, mania, anxiety, or depression, as well as other drug dependence or abuse (Sabe et al., 2019). Additionally, observational data have indicated an association between non-benzodiazepine sedative hypnotics and fracture risk (Wang et al., 2001; Finkle et al., 2011). However, there is limited data on their safety in elderly patients, especially regarding posture instability, falls, and fractures (Nishtala and Chyou, 2017).

### 3.4 Full agonists and partial agonists

**FIGURE 2:**
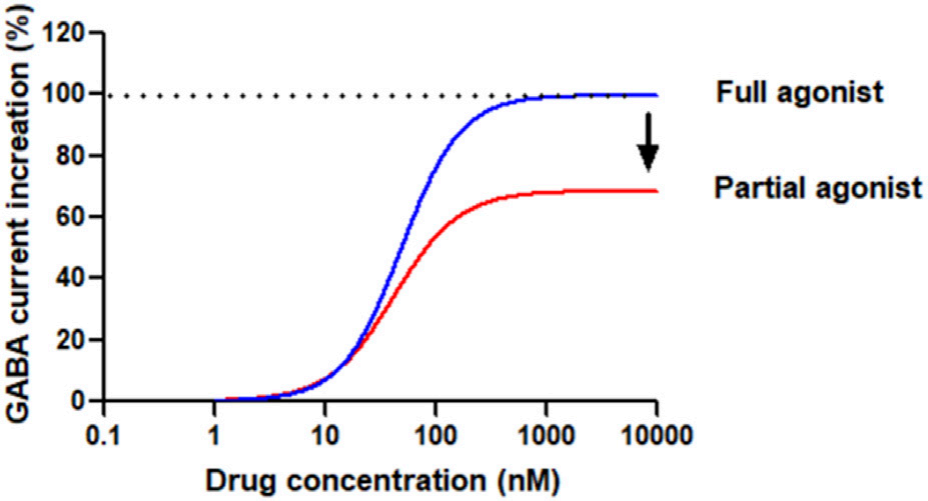
Conceptual diagram of compounds bound to the benzodiazepine site of GABAAR as agonists. Full agonists, such as Diazepam or Lorazepam, have a high (* 100%) intrinsic effect on receptors, increasing GABA inhibitory activity in a dose-dependent manner until maximum activity is reached. The dose-response curve is steep, and the maximum effect is achieved at relatively low drug concentrations based on the studied effects. Partial agonists, based on the intrinsic efficacy, also increase the inhibitory activity of GABA in a dose-dependent manner, but cannot achieve the maximum effect of a full agonist. The dose-response curve of some agonists is flat and can only reach peak effects even the drug concentration is very high.

Although BZDs and nBZDs have quick effects, they are primarily used as short-term treatments; long-term or high-dose use of these drugs will lead to tolerance, rebound insomnia after drug withdrawal, and increased risks of abuse and addiction (Lugoboni et al., 2014). The ideal hypnotic drug should feature the following characteristics: fast absorption/action, ideal hypnotic effect, improving abnormal sleep phase without affecting physiological sleep, maintaining sufficient sleep time, fast elimination, no accumulation, no drug hangover after waking up, however so far there is no drug fully meet these requirements. Therefore, there are considerable unmet medical needs to develop novel compounds for insomnia that lack these side effects. The development of partial agonists targeting benzodiazepine sites is one of the strategies to solve the problem of BZD receptor ligand tolerance and dependence (Stephens and Sarter, 1988). The general concept of full and partial BZD agonists is shown in Figure 2.

From a therapeutic viewpoint, the introduction of full GABAAR agonists as drugs presents challenges. Full agonists cause rapid desensitization of synaptic GABAARs. Partial agonists, however, offer a distinct mechanism by eliciting varying receptor responses based on the existing activation level of the GABAARs. As the concentration of the partial agonist rises, it progressively displaces GABA, establishing a steady activation level that reflects its own efficacy. This shift results in the transition from phasic receptor activation by GABA on synaptic receptors to a more tonic activation pattern. Consequently, depending on the partial agonist’s efficacy, there may be some level of desensitization. The functional analysis of partial agonists has historically been challenging, often yielding inconsistent results across different tissues for the same ligands. It is crucial to have homogeneous receptor populations to accurately comprehend structure-activity relationships (SARs) and distinguish between partial and full agonism. 5-(4-piperidyl)-3-hydroxyisoxazole (4-PIOL), a low-efficacy partial agonist, for instance, exhibits limited capacity to maintain the ion channel in an open state, only enabling brief channel openings. The conductance of channels opened by 4-PIOL is identical to that of GABA, indicating a similar conformation of the open states. These findings were later confirmed and broadened when a correlation between efficacy and channel open duration (with unchanged conductance) was observed for various GABAAR ligands with efficacies from low (4-PIOL) to full agonism in recombinant α1β3γ2 GABAARs (Mortensen et al., 2004). Replacing the 3-hydroxyisoxazole ring of 4-PIOL with a 3-hydroxyisothiazole ring to create thio-4-PIOL results in a higher, yet still low, efficacy due to slightly longer channel open durations in recombinant α1β3γ2 GABAARs. Additionally, thio-4-PIOL shows subtype selectivity, with greater efficacy for extrasynaptic subtypes (α5β2/3γ2, α4β2/3δ, α6β2/3δ) of GABAARs, particularly in the presence of β3, compared to synaptic types (α1β2/3γ2, α2β2/3γ2, α3β2/3γ2), where it induces only low activity (Krall et al., 2015; Hoestgaard-Jensen et al., 2013).

## 4 The recently approved novel partial benzodiazepine receptor agonist

The recently approved Dimdazenil by the NMPA of China is a first-in-class drug developed by Zhejiang Jingxin Pharmaceutical Co., Ltd. in China under the license of Evotec for the treatment of insomnia. The first partial positive allosteric modulator of GABAAR at the benzodiazepine site, also called benzodiazepine receptor partial agonists may offer better clinical performance, which clinical data collected to date indicated that it avoids some of the side effects caused by excessive enhancement of GABAARs produced by “complete” or “super” agonists such as Zolpidem and Dexzopiclone, which were approved in China in 1995 and 2007 respectively (Huang et al., 2024).

### 4.1 Mechanism of action

*In vitro* pharmacological trials have shown that Dimdazenil acts as a partial agonist on the benzodiazepine-GABAAR complex (Huang et al., 2024; Wang et al., 2023). The results showed that Dimdazenil has a high affinity to the GABAAR. As a partial positive allosteric modulator of GABAAR, Dimdazenil has been shown to facilitate GABA currents by mild allosteric excitation. Compared to GABAARs containing α2 and α3 subunits, Dimdazenil is only moderately selective (∼3 to 4 fold) to GABAARs containing α1 subunits (Huang et al., 2024; Wang et al., 2023).

### 4.2 Pharmacokinetic characteristics and comparison

The pharmacokinetic profile of Dimdazenil highlights its promise as a BZR partial agonist. It reaches maximum plasma concentration (Tmax) in approximately 1 h, facilitating a rapid onset of action relative to full agonist medications (Wang et al., 2023). Dimdazenil also exhibits a longer elimination half-life of 4 h compared to short-acting BZR full agonists such as Triazolam, which have an optimal dose half-life of 1.5–3.5 h, thereby ensuring prolonged sleep maintenance. Additionally, Dimdazenil has a reduced risk of residual effects when compared to intermediate and long-acting BZR full agonists like Lorazepam, Clonazepam, and Diazepam, which possess average half-lives exceeding 8 h (Kienitz et al., 2022; Wang et al., 2023).

### 4.3 Efficacy, safety, and potential benefits

In clinical efficacy and safety studies, Dimdazenil 1.5, 2.5, and 5 mg improved certain objective and subjective sleep outcomes in insomnia patients compared with placebo (Huang et al., 2024; Li et al., 2024), and Dimdazenil 2.5 mg had significant benefits on sleep maintenance and sleep onset in insomnia patients, with favorable safety and tolerability. More importantly, it doesn’t affect daytime functioning (Huang et al., 2024). In phase II and III clinical trials, Dimdazenil appears to extend total sleep duration and increase the proportion of time spent in stage 2 sleep while also prolonging the latency to REM sleep. Conversely, it seems to reduce the percentage of time spent in REM sleep and the duration of stage 3 sleep, with these effects being dose-dependent (Huang et al., 2024; Li et al., 2024). The clinical efficacy of Dimdazenil supports its continued development for additional insomnia disorders, with significant improvements in sleep initiation, sleep maintenance, and sleep depth/quality. The drug was well tolerated with no sedation or withdrawal symptoms after withdrawal. Further, Dimdazenil has no deleterious effects on cognitive function in clinical trials (Huang et al., 2024). Most of AEs are mild, transient, and do not cause treatment interruption. So far, no major safety signals or issues have been identified. Based on current clinical data, as shown in the Table 1, the side effects of Dimdazenil appear to be more favorable compared to full GABAA receptor agonists, such as benzodiazepines and provides a much-anticipated new addition for the treatment of insomnia disorder, a widespread condition with limited effective and safe treatments.

**TABLE 1:** Comparison of classic benzodiazepines, non-benzodiazepines, and novel partial benzodiazepine drugs for insomnia.

Mechanism	Categories	Drug	Structure	Recommended dosage	Characteristics	Weaknesses or risk
BZR partial agonists	Novel benzodiazapines	Dimdazenil	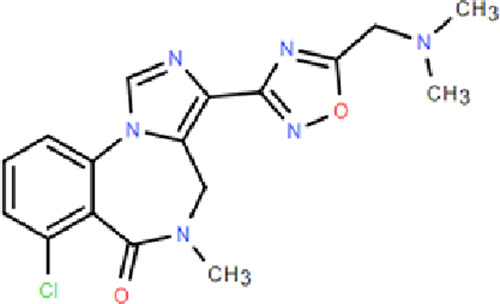	2.5 mg	Rapid onset; Sleep maintenance; Low risk of drug-drug interaction; Low risk of drug addiction; Significant easing effect on anxiety and tension	—
BZR full agonists	Benzodiazepines	Diazepam	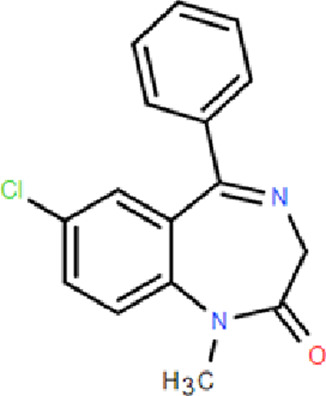	2.5–5 mg	Long half-life; Rapid onset	Sedation, ataxia, and memory impairment; Continued use will develop tolerance, and withdrawal symptoms will occur after stopping treatment; Residual effects, respiratory depression and drug resistance, addiction, and other problems Ebert et al. (2006); Lader (2011)
		Lorazepam	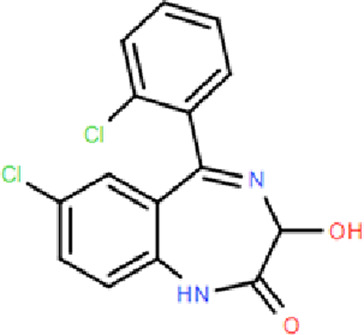	2–3 mg	Medium half-life; Rapid onset, significant easing effect on anxiety and tension	
		Clonazepam	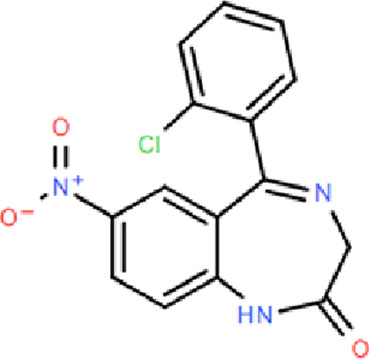	1–4 mg	Long half-life; Improving difficulty falling asleep and reducing daytime sleepiness	
		Triazolam	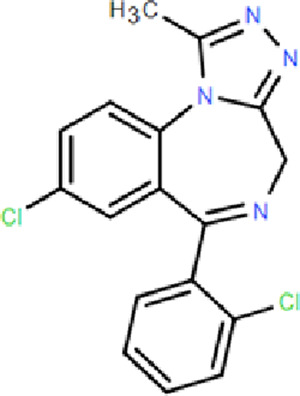	0.25–0.5 mg	Short half-life; Rapid onset	
	Non-benzodiazepine	Zolpidem	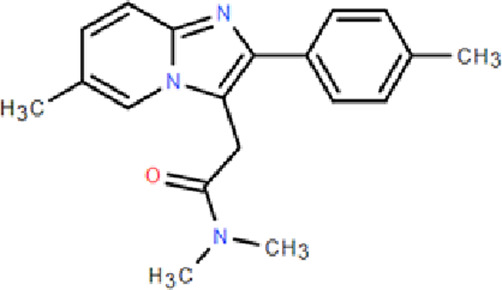	10 mg	Reducing sleep latency; Improving total sleep time; Reducing nighttime awakenings	May cause drowsiness, dizziness, and impaired coordination; Long-term use may lead to tolerance, dependence, and withdrawal symptoms upon discontinuation Ebert et al. (2006)
		Zopiclone	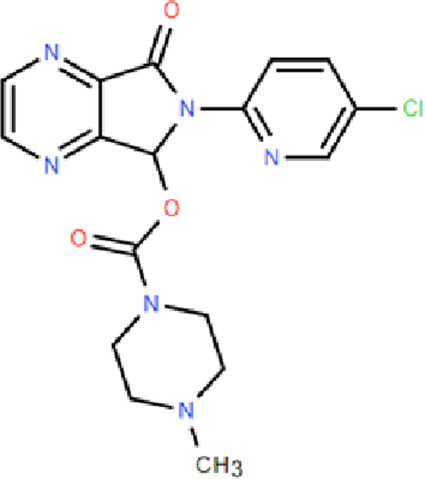	7.5 mg	Decreasing sleep latency and the number of night-time awakenings; Increasing sleep duration	Showing residual effects of poor driving performance next-day Luzzi et al. (2022)
		Eszopiclone	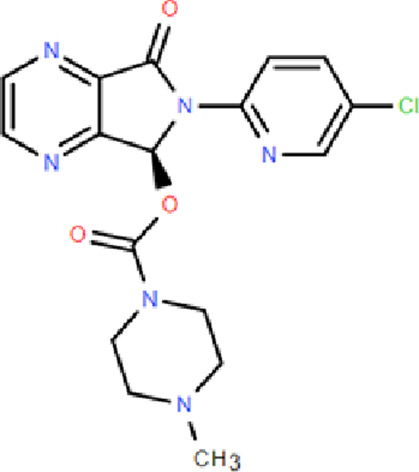	2–3 mg	Reducing the time to onset of sleep; Increasing total sleep time	May cause daytime drowsiness, dizziness, and impaired cognitive function
		Zaleplon	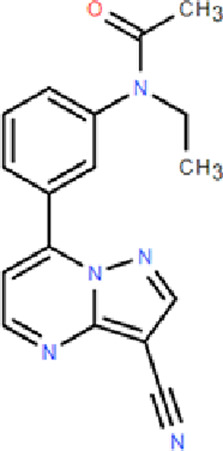	5–10 mg	Rapid onset	Not for patients with sleep maintenance disorders; Affecting next day performance Ebert et al. (2006)

## 5 Conclusion

Starting from the early sedative-hypnotic drugs, great progress has been made in the development of effective drugs for the treatment of insomnia especially with the emergence of benzodiazepines and non-benzodiazepines. However, with the increasing application of traditional benzodiazepines and non-benzodiazepines, adverse reactions such as dependence, tolerance, and other problems started to impact patients’ quality of life which demanded the development of safer and better tolerated drugs. The approval of Dimdazenil, the first partial positive allosteric modulator of GABAAR brings more options to the patients and hopefully inspires more novel approaches to solve the unmet medical needs of insomnia patients.
